# SR-A ligand and M-CSF dynamically regulate SR-A expression and function in primary macrophages via p38 MAPK activation

**DOI:** 10.1186/1471-2172-12-37

**Published:** 2011-07-07

**Authors:** Dejan Nikolic, Lindsay Calderon, Liqin Du, Steven R Post

**Affiliations:** 1 Department of Pathology, University of Arkansas for Medical Sciences, Little Rock, Arkansas, 72205 USA; 2 Department of Molecular and Biomedical Pharmacology, University of Kentucky, Lexington, Kentucky, 40536 USA; 3 Harold C. Simmons Comprehensive Cancer Center, University of Texas Southwestern Medical Center, Dallas, Texas, 75390 USA

## Abstract

**Background:**

Inflammation is characterized by dynamic changes in the expression of cytokines, such as M-CSF, and modifications of lipids and proteins that result in the formation of ligands for Class A Scavenger Receptors (SR-A). These changes are associated with altered SR-A expression in macrophages; however, the intracellular signal pathways involved and the extent to which SR-A ligands regulate SR-A expression are not well defined. To address these questions, SR-A expression and function were examined in resident mouse peritoneal macrophages incubated with M-CSF or the selective SR-A ligand acetylated-LDL (AcLDL).

**Results:**

M-CSF increased SR-A expression and function, and required the specific activation of p38 MAPK, but not ERK1/2 or JNK. Increased SR-A expression and function returned to basal levels 72 hours after removing M-CSF. We next determined whether prolonged incubation of macrophages with SR-A ligand alters SR-A expression. In contrast to most receptors, which are down-regulated by chronic exposure to ligand, SR-A expression was reversibly increased by incubating macrophages with AcLDL. AcLDL activated p38 in wild-type macrophages but not in SR-A-/- macrophages, and p38 activation was specifically required for AcLDL-induced SR-A expression.

**Conclusions:**

These results demonstrate that in resident macrophages SR-A expression and function can be dynamically regulated by changes in the macrophage microenvironment that are typical of inflammatory processes. In particular, our results indicate a previously unrecognized role for ligand binding to SR-A in up-regulating SR-A expression and activating p38 MAPK. In this way, SR-A may modulate inflammatory responses by enhancing macrophage uptake of modified protein/lipid, bacteria, and cell debris; and by regulating the production of inflammatory cytokines, growth factors, and proteolytic enzymes.

## Background

SR-A is a multifunctional macrophage receptor that is upregulated during monocyte differentiation into macrophages, and is further increased in pathological conditions such as diabetes [1-3]. SR-A is also highly expressed by macrophages in atherosclerotic lesions and Alzheimer's plaques [4-6]. In contrast, decreased SR-A expression is associated with increased susceptibility to bacterial infection, progression of prostate cancer, and enhanced cytokine production [7-9]. Such results suggest important and complex roles for SR-A in modulating immune function and inflammation.

Macrophage differentiation and recruitment during inflammation is mediated by changes in the local environment and the secretion of cytokines/chemokines such as M-CSF. M-CSF, which is produced by many cell types, is a cytokine that plays an essential role in monocyte-macrophage functions including membrane ruffling, cell migration, and the production of inflammatory mediators [reviewed in [10,11]]. Because of its role in the development of monocyte/macrophage cells, M-CSF is thought to play important roles in immune function and inflammatory diseases [reviewed in [12]]. For example, M-CSF is thought to promote atherosclerosis by increasing macrophage viability/differentiation, low-density lipoprotein (LDL) receptor-mediated lipoprotein uptake, and the expression of macrophage SR-A [12-14]. SR-A promotes foam cell formation by binding and internalizing modified lipoproteins [e.g., acetylated LDL (AcLDL), oxidized LDL (oxLDL)], but not native lipoproteins [15]. SR-A has also been associated with additional macrophage functions including cell adhesion to modified extracellular matrix, phagocytosis/clearance of apoptotic cells, and the modulation of macrophage activation and cytokine production [16-20]. Thus, increased SR-A expression may play an important part in the effects of M-CSF on immune function and inflammation. Taken further, the ability to inhibit M-CSF-induced SR-A expression may have important therapeutic implications. However, the cellular pathways that couple M-CSF binding to increased SR-A expression are not known.

In addition to increased secretion of M-CSF, local inflammation results in the modification of proteins, alterations in the extracellular matrix (ECM), and tissue damage. Such modifications result in the formation and accumulation of SR-A ligands. Many receptors including receptor-tyrosine kinases (e.g., insulin receptors), G protein-coupled receptors (e.g., β-adrenergic receptors), and nutrient receptors (e.g., LDL receptors) are down-regulated by prolonged exposure to ligand. This negative feedback is mediated by activation of intracellular signaling pathways that regulate receptor expression. Although it might be of particular importance in diverse inflammatory conditions, the effect of modulating the concentration of SR-A ligand in tissue on SR-A expression in resident macrophages is not known.

SR-A gene expression is under the control of a proximal promoter in combination with an upstream enhancer element [2,21,22]. Binding of the transcription factor AP-1 to this upstream enhancer element has been shown to be sufficient to direct specific macrophage SR-A expression [1,22]. Activation of AP-1 in inflammatory cells is primarily regulated by the mitogen activated protein kinases (MAPK), in particular via c-Jun phosphorylation by JNK and ATF2 phosphorylation by p38 MAPK [23]. Roles for both of these MAPKs in regulating SR-A expression in elicited macrophages has been suggested [24,25].

It has been suggested that different agents used to elicit resident peritoneal macrophages can alter macrophage populations, their regulation by intracellular signals, and macrophage responses e.g., superoxide production, chemokine generation, and Ab-dependent cell-mediated cytolysis [26,27]. In this study, we used isolated resident peritoneal macrophages to examine the intracellular signaling pathways involved in regulating SR-A expression and function. We also examined whether chronic exposure to SR-A ligand alters SR-A expression in macrophages. Our results demonstrate that cytokine and SR-A ligand reversibly enhance SR-A expression and function via activation of p38 MAPK and the subsequent induction of SR-A transcription.

## Methods

### Chemicals

Dulbecco's modified Eagle's medium (DMEM) supplemented with L-glutamine, DMEM with 25 mM HEPES, and heat-inactivated fetal bovine serum (FBS) were purchased from GibcoBRL (Grand Island, NY). Penicillin and streptomycin, fucoidan, and actinomycin D were purchased from Sigma (St Louis, MO). Recombinant murine M-CSF and goat anti-mouse SR-A antibody were purchased from R&D Systems (Minneapolis, MN). Rabbit anti-phospho-p44/42 (ERK1/2), anti-phospho-p38, anti-phospho-SAPK/JNK, and anti-p38 MAPK antibodies were purchased from Cell Signaling Technology, Inc. (Beverly, MA). Anti-p44/42 (ERK1/2) and anti-JNK1 antibodies were purchased from Santa Cruz Biotechnology, Inc (Santa Cruz, CA). The specific MAPK inhibitors SB203580 (p38), SP600125 (JNK1/2), and PD98059 (MEK) were purchased from EMD Biosciences, Inc (La Jolla, CA). Alexa^488^-Acetylated LDL was purchased from Molecular Probes, Inc (Eugene, OR) and Alexa^647^-conjugated 2F8 mAb from Serotec.

### Cell culture and treatment

**Figure 1:**
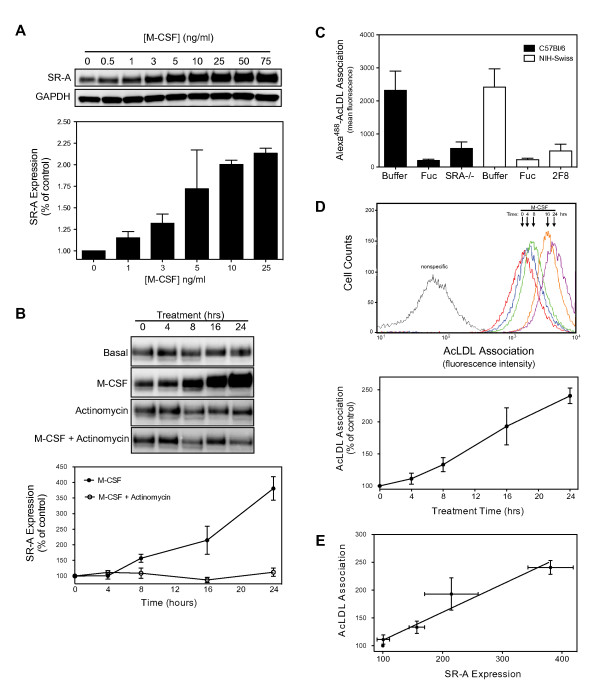
**M-CSF induces SR-A expression and AcLDL association**. (*A*) Cultured resident MPMs were incubated with the indicated concentrations of M-CSF for 24 hrs. Subsequently, SR-A and GAPDH proteins were detected in lysates prepared from treated cells by immunoblotting with specific antibodies. A representative immunoblot from a single experiment and the results (mean ± SEM) from at least three separate experiments are shown. (*B*) Cultured resident MPMs were pretreated with actinomycin D (5 μM) for 30 min, and then incubated with or without M-CSF (25 ng/ml) for the indicated times prior to assessing SR-A expression. A representative immunoblot from a single experiment and the results (mean ± SEM) from at least three separate experiments are shown. (*C*) Alexa^488^-AcLDL association with macrophages isolated from C57Bl/6 or NIH-Swiss mice was quantified by flow cytometry as described in *Material and Methods*. Non-specific association was determined in the presence of fucoidan (Fuc, 75 μg/ml), a blocking SR-A antibody (2F8, 10 μg/ml), or by using SR-A-/- macrophages. Shown is the mean ± SEM of three separate experiments. (*D*) SR-A-specific Alexa^488^-AcLDL association with macrophages incubated with M-CSF (25 μg/ml) for the indicated times was quantified by flow cytometry as described in *Material and Methods*. The results of a representative experiment and the mean ± SEM of at least three separate experiments are shown. (*E*) Relative changes in SR-A expression and function were plotted and the best fit line determined using a Deming linear regression model in GraphPad Prism.

Resident (non-elicited) mouse peritoneal macrophages (MPMs) were harvested by peritoneal lavage with ice-cold sterile saline from male NIH Swiss mice (Harlan; Indianapolis, IN), and from C57Bl/6 and SR-A-/- mice on a C57Bl/6 background (Figures 1C and 4B; Jackson Laboratory, Bar Harbor, ME). Animal care and use for all procedures were done according to protocols reviewed and approved by the Institutional Animal Care and Use Committee at University of Arkansas for Medical Sciences and the University of Kentucky. Isolated macrophages were cultured as previously described [28]. Briefly, peritoneal exudates were incubated overnight at 37°C and non-adherent cells removed by gently washing with PBS. Adherent macrophages were maintained in DMEM containing antibiotics and FBS (10%) for 48 hrs prior to use in experiments. MPMs were then treated with agonists in the presence or absence of specific inhibitors as described in figure legends. Following treatment, MPMs were washed with ice-cold PBS and cell lysates prepared by incubating cells in MBST/OG buffer (25 mM MES; 150 mM NaCl; 60 mM octylglucopyranoside; 1% Triton X-100; pH 6.4) containing protease and phosphatase inhibitors (Sigma, St. Louis, MO) for 45 min on ice. Cell lysates were centrifuged at 13,000 rpm for 15 min and the pellets were discarded. Protein concentration of supernatant was determined using the BioRad DC assay using BSA as a standard (Hercules, CA).

### Western blot analysis

Equal amounts of cell lysate protein were resolved by 12% SDS-PAGE and transferred to polyvinylidene difluoride (PVDF) membrane (Millipore, Billerica, MA). Proteins were detected by immunoblotting with specific primary antibodies followed by incubation with species-specific HRP-conjugated secondary antibodies. Bands were visualized by chemiluminescence, images captured with a Kodak Image Station 4000 MM Pro, and band intensities quantified using Kodak 1D image analysis software.

### Flow Cytometry

To assess SR-A-mediated lipoprotein uptake and surface accessible SR-A, cultured MPMs were preincubated for 2 hrs in serum-free DMEM and then incubated for 2 hrs at 37°C with Alexa^488^-AcLDL(2.5 μg/ml). Nonspecific association of Alexa^488^-AcLDL with cells was defined in the presence of the SR-A ligand fucoidan (75 μg/ml) for 5 minutes prior to addition of lipoprotein. Nonspecific values were subtracted from total values to calculate SR-A-specific cell association. To quantify surface SR-A, MPMs were washed and incubated in DMEM/1% FBS containing Alexa^647^-conjugated 2F8 mAb for 30 min at 25°C. Cells were then washed and suspended in PBS, and Alexa^488^-AcLDL and Alexa^647^-conjugated antibody association quantified by flow cytometry.

## Results and Discussion

### M-CSF induces SR-A expression and AcLDL association

Macrophage differentiation and recruitment into inflammatory sites are associated with increased SR-A expression. SR-A expression is regulated by both transcriptional and post-transcriptional processing [1,3,14,25,28-30]. M-CSF is involved in both monocyte/macrophage differentiation and recruitment during inflammation, and has previously been shown to enhance SR-A expression in elicited macrophages via increased transcription [14]. However, the intracellular signals that couple M-CSF to enhanced SR-A expression have not been defined.

To examine the signaling pathways that regulate SR-A expression, the effect of M-CSF on SR-A expression was examined in non-elicited, resident MPMs. Non-elicited, resident MPMs were used because of the potential for eliciting agents to alter macrophage phenotype and regulation by intracellular signals. Culturing isolated MPMs with M-CSF resulted in the concentration-(Figure 1A) and time-(Figure 1B) dependent induction of SR-A protein expression. SR-A expression was increased by incubating macrophages with physiologically relevant (0.5-75 ng/ml) concentrations [31-33] of M-CSF (EC_50 _≈5 ng/ml), and was maximally induced following a 24 hr incubation with M-CSF (Figure 1B). No further increase in expression was observed with longer times of incubation with M-CSF (data not shown). Treating MPMs with actinomycin D (5 μM), an inhibitor of gene transcription, prior to incubation with M-CSF abolished M-CSF-mediated upregulation of SR-A expression indicating that M-CSF-stimulated SR-A expression in resident macrophages results from increased SR-A transcription. To confirm that increased SR-A expression is correlated with enhanced SR-A function, we examined the ability of M-CSF to stimulate fluorescently-labeled AcLDL (Alexa^488^-AcLDL) association with MPMs. Results shown in Figure 1C show that a blocking SR-A monoclonal antibody (2F8) and an excess of SR-A competitor (fucoidan) reduced AcLDL association with macrophages isolated from C57Bl/6 or NIH-Swiss mice to a level similar to that obtained using macrophages isolated from SR-A-/- in a C57Bl/6 background. These results demonstrate the specificity of this asssay for assessing SR-A function. M-CSF treatment induced a time-dependent increase in AcLDL association with MPMs that was maximal (2.4-fold) at 24 hrs (Figure 1D), with no further increase at longer incubation times (data not shown). M-CSF stimulation of SR-A function was linearly correlated (Pearson r = 0.97; p = 0.007) with the effect of M-CSF on SR-A expression (Figure 1E) indicating that in resident MPMs SR-A function is limited, at least in part, by the level of receptor expression. These results suggest that by increasing SR-A expression M-CSF, produced for example by endothelial cells or lymphocytes in an atherosclerotic plaque or other inflammatory sites, can increase the uptake of modified lipoprotein and other scavenger receptor ligands.

### M-CSF-stimulates SR-A expression by upregulating SR-A mRNA and protein synthesis which requires p38 MAPK activation

**Figure 2:**
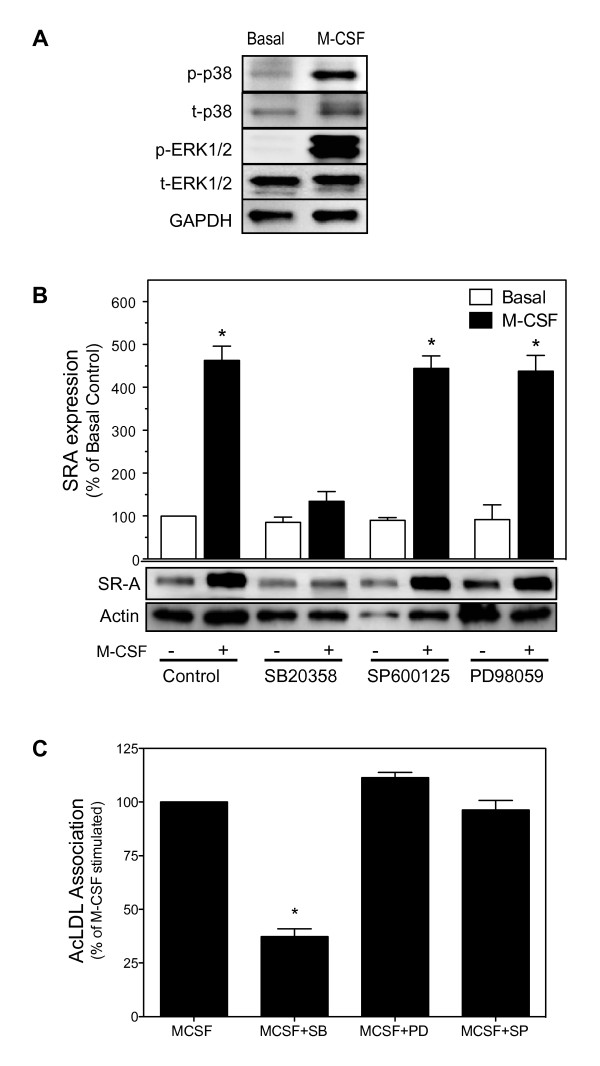
**M-CSF-stimulates SR-A expression by upregulating SR-A mRNA and protein synthesis which requires p38 MAPK activation**. (*A*) MPM were incubated with or without M-CSF (25 ng/ml) for 10 min at 37°C. Cells were then lysed with MBST/OG buffer and phosphorylation of MAPK quantified by immunobloting. The results from a single experiment are shown and are representative of at least four separate experiments. (*B*) MPM were treated as indicated with specific inhibitors of p38 [SB203580 (10 μM)], JNK [SP600125 (20 μM)] or ERK1/2 [PD98059 (10 μM)] for 20 minutes at 37°C. Cells were then treated with or without M-CSF (25 ng/ml) for 24 hrs at 37°C and SR-A protein quantified by immunoblotting. Data represent the mean ± SEM from at least three independent experiments.* denotes significant difference (p < 0.05) from untreated control value (ANOVA with Dunnett's multiple comparison). (*C*) MPM were treated with specific inhibitors of p38 [SB203580 (10 μM)], JNK [SP600125 (20 μM)] or ERK1/2 [PD98059 (10 μM)] for 20 minutes at 37°C, and then incubated with M-CSF (25 ng/ml) for 24 hrs. SR-A-specific macrophage association was quantified by flow cytometry as described in *Materials and Methods*. Data represent the mean ± SEM from at least three separate experiments. * denotes significant difference (p < 0.05) from M-CSF treated control value (ANOVA with Dunnett's multiple comparison).

Many effects of M-CSF including macrophage migration, differentiation, survival, and cytokine production are mediated, in part, via activation of MAPKs, a family of kinases that include ERK1/2, JNK, and p38 [34-36]. Activation of MAPKs, in particular JNK and p38 MAPK, regulates the activity of several transcription factors including AP-1 [23]. The binding of AP-1 to an upstream enhancer element is sufficient to direct specific macrophage SR-A expression in inflammatory cells [1,21,22]. Therefore, we examined M-CSF-dependent MAPK activation and whether MAPK activation was required for M-CSF-induced SR-A expression. For this, resident MPMs were treated with M-CSF and the activation of ERK, p38, and JNK assessed by immunoblotting with phospho-specific MAPK antibodies. As shown in Figure 2A, M-CSF induced the phosphorylation of both p38 and ERK1/2. In contrast, JNK phosphorylation was not detectable in either the presence or absence of M-CSF (data not shown). To determine if activation of MAPK was specifically required for M-CSF-induced SR-A expression and function, the ability of M-CSF to stimulate SR-A expression and AcLDL association was assessed in MPMs pretreated with specific inhibitors of p38 MAPK (SB203580), JNK (SP600125), and MEK1 (PD98059), which inhibits ERK1/2 activation. Inhibiting JNK or ERK1/2 activation had no effect on either M-CSF-induced SR-A expression (Figure 2B) or M-CSF-induced uptake of modified lipoprotein (Figure 2C). In contrast, pretreating macrophages with SB203580 inhibited both M-CSF-induced SR-A expression and modified lipoprotein uptake (Figure 2C). Together, these data define a specific requirement for activation of p38 MAPK, but not ERK1/2 and JNK, in M-CSF-induced SR-A expression and function.

### M-CSF stimulates SR-A expression and AcLDL association in reversible manner

**Figure 3:**
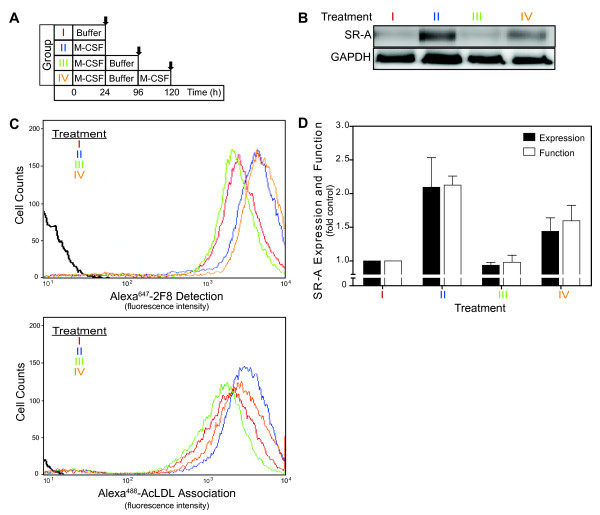
**M-CSF reversibly stimulates SR-A expression and AcLDL association**. (*A*) Incubation scheme: Cultured resident MPM were incubated (I) without M-CSF; (II) with M-CSF (25 ng/ml) for 24 hrs; (III) with M-CSF (25 ng/ml) for 24 hrs and then in absence of M-CSF for additional 72 hrs; or (IV) with M-CSF (25 ng/ml) for 24 hrs, then in absence of M-CSF for additional 72 hrs, and incubated again with M-CSF (25 ng/ml) for 24 hrs. SR-A expression and function were assessed at the end of incubation (arrows). (*B*) Following incubations, cell lysates were prepared and SR-A protein was quantified by immunoblotting. (*C*) Following incubations, surface SR-A (top) and SR-A-mediated uptake (bottom) were quantified by flow cytometry using Alexa^647^-conjugated 2F8 mAb and Alexa^488^-AcLDL as described in *Materials and Methods*. The results of representative experiments are shown. (*D*) The mean ± SEM of at least three separate flow cytometry experiments are shown.

Increased SR-A expression and function following M-CSF treatment suggests that regulating SR-A expression in resident macrophages is an adaptive response to changes in the local inflammatory environment. Inflammation is a dynamic process in which the production of cytokines such as M-CSF changes as inflammation resolves over time. To test whether the enhanced SR-A expression and function induced by M-CSF is reversible, we examined SR-A protein and AcLDL uptake following removal of M-CSF using the incubation scheme depicted in Figure 3A. The increase in SR-A expression, as quantified by western blotting (Figure 3B) or flow cytometry (Figure 3C**top**), and function (Figure 3C**bottom**) observed following M-CSF treatment returned to the pretreated levels 72 hr after M-CSF removal (t 1/2 ≈ 24 hr). To determine if SR-A expression was still responsive to M-CSF, previously treated MPMs were re-stimulated with M-CSF for another 30 hrs. Similar to naïve MPMs, SR-A expression and function were increased by the restimulation with M-CSF. As summarized in Figure 3D, M-CSF-induced proportional changes in SR-A expression and function in both naïve and previously treated MPMs. Together, these data demonstrate that in resident macrophages SR-A expression and function is dynamically regulated by M-CSF.

### AcLDL stimulates SR-A expression in reversible manner via activation of p38 MAPK

**Figure 4:**
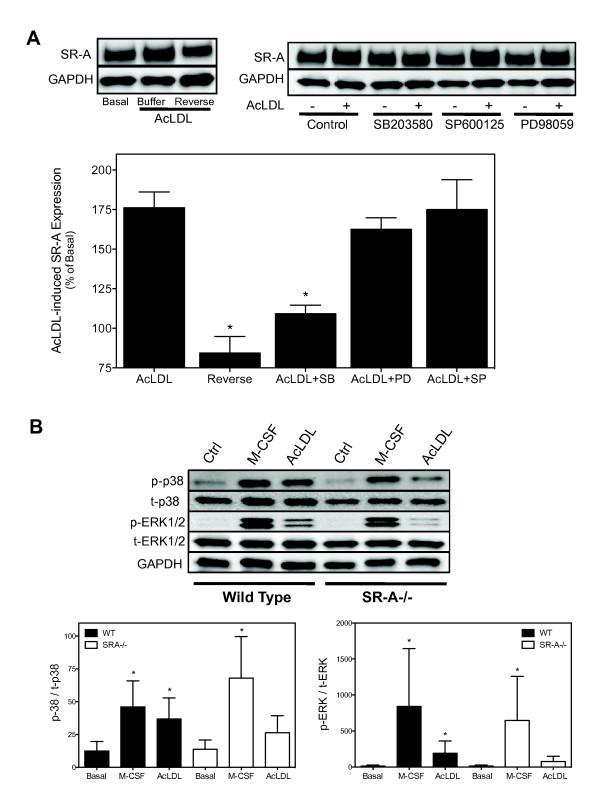
**AcLDL reversibly stimulates SR-A expression via activation of p38 MAPK**. (*A*) Cultured resident MPM were pretreated, as indicated, with specific inhibitors of p38 [SB203580 (10 μM)], JNK [SP600125 (20 μM)] or ERK1/2 [PD98059 (10 μM)] for 20 minutes at 37°C. Cells were then treated with or without AcLDL (50 μg/ml) for 24 hrs at 37°C, and as indicated incubated without AcLDL for additional 72 hrs (Reverse). Cell lysates were prepared and SR-A protein expression quantified by immunoblotting. A representative immunoblot from a single experiment and the results (mean ± SEM) from at least three separate experiments are shown. * denotes significant difference (p < 0.05) from AcLDL treated control value (ANOVA with Dunnett's multiple comparison). *(B*) To determine whether AcLDL induces MAPK activation in SR-A dependent manner, MPM from wild type and SR-A-/- mice were cultured for 48 hrs and then incubated without or with M-CSF (25 ng/ml) or AcLDL (50 μg/ml) for 10 min. Cells lysates were prepared and phosphorylation of individual MAPKs detected by immunoblotting. A representative immunoblot from a single experiment and the results (mean ± SEM) from at least three separate experiments are shown. * denotes significant difference (p < 0.05) from untreated wild-type value (ANOVA with Dunnett's multiple comparison).

In addition to cytokines, inflammatory settings are characterized by accumulation of SR-A ligands including oxidized lipoproteins (e.g., modified LDL), necrotic cell debris, and modified ECM. Because many receptors are down-regulated by chronic exposure to ligand, we tested whether SR-A expression was decreased by ligand. In contrast to our hypothesis, incubating macrophages with an SR-A selective ligand (AcLDL; 50 μg/ml) for 24 hr increased SR-A protein expression (Figure 4A). As demonstrated for M-CSF, enhanced SR-A expression returned to the pretreated levels 72 hr after AcLDL was removed, and pre-treating MPMs with a specific p38 MAPK inhibitor (SB203580) blocked AcLDL-induced SR-A expression. In contrast, inhibitors of ERK1/2 and JNK did not affect the ability of AcLDL to enhance SR-A expression. To confirm that incubating resident MPMs with AcLDL induced SR-A-dependent MAPK activation, the ability of AcLDL to activate MAPK in wild-type and SR-A deficient resident MPMs was examined. As shown in Figure 4B, treating macrophages with AcLDL (50 μg/ml, 10 min) induced phosphorylation of p38 MAPK and ERK1/2 in wild-type but not in SR-A deficient macrophages. In contrast, p38 MAPK and ERK1/2 phosphorylation were similarly increased in both wild type and SR-A deficient MPMs treated with M-CSF. JNK phosphorylation was not detected in any treatment group (data not shown). Together, these data indicate that ligand binding to SR-A is positively coupled to SR-A expression via specifically activating p38 MAPK.

Regulating SR-A expression in vivo may be relevant to many inflammatory disorders. For example, M-CSF plays important roles in inflammation and immunity. M-CSF increases anti-tumor and anti-infective functions of macrophages, whereas M-CSF deficiency decreases atherosclerosis and impairs osteoclast development [11,37]. The extent to which these effects depend on altered SR-A expression remains to be defined. However, the possibility that regulating SR-A expression modulates inflammatory responses is suggested by the many studies showing that decreased SR-A expression is associated with reduced atherosclerosis, increased susceptibility to infection, disease progression in prostate cancer, and dysregulation of bone development [8,9,38-40]. Like SR-A, activation of p38 MAPK is important for many inflammatory processes including the production of TNFα, IL-1β, and other cytokines [41,42]. In addition, inhibiting p38 MAPK has shown potential benefit in the treatment of inflammatory disease [43,44]. Our findings suggest that the effects of M-CSF and p38 MAPK on immune and inflammatory processes may be mediated, in part, by modulating SR-A expression.

## Conclusions

Our results indicate that resident tissue macrophages adapt to changes in their local environment by modulating SR-A expression and function. Such modulation may involve the local secretion of M-CSF, which increases SR-A expression via activating p38 MAPK. Unlike many receptor systems which are down-regulated by ligand, ligand binding to SR-A up-regulates SR-A expression by activating p38 MAPK. Increased SR-A expression might modulate inflammation by enhancing macrophage uptake and clearance of modified protein/lipid, bacteria, and cell debris. In addition, SR-A-mediated p38 MAPK activation may regulate the production of inflammatory cytokines, growth factors, and proteolytic enzymes, and therefore modulate the progression of many inflammatory disorders [18-20]. This may be of particular importance in settings where SR-A ligands accumulate such as atherosclerosis, diabetes, and Alzheimer's disease.

## Competing interests

The authors declare that they have no competing interests.

## Authors' contributions

DN contributed to performing and analyzing experiments, and in preparing manuscript. LC and LD contributed to performing and analyzing experiments. SRP contributed to experimental design, interpretation of results, and manuscript preparation. All authors read and approved the final manuscript
